# Tixagevimab/Cilgavimab as Pre-Exposure Prophylaxis against COVID-19 for Multiple Myeloma Patients: A Prospective Study in the Omicron Era

**DOI:** 10.3390/diseases11030123

**Published:** 2023-09-18

**Authors:** Ioannis Ntanasis-Stathopoulos, Charalampos Filippatos, Maria Gavriatopoulou, Panagiotis Malandrakis, Evangelos Eleutherakis-Papaiakovou, Vassiliki Spiliopoulou, Rodanthi-Eleni Syrigou, Foteini Theodorakakou, Despina Fotiou, Magdalini Migkou, Maria Roussou, Efstathios Kastritis, Meletios Athanasios Dimopoulos, Evangelos Terpos

**Affiliations:** Department of Clinical Therapeutics, School of Medicine, National and Kapodistrian University of Athens, 11528 Athens, Greece; johnntanasis@med.uoa.gr (I.N.-S.); harry.filippatos@gmail.com (C.F.);

**Keywords:** Evusheld, COVID-19, SARS-CoV-2, pre-exposure prophylaxis, multiple myeloma, hematological malignancies, antibodies

## Abstract

**Background:** tixagevimab/cilgavimab, distributed under the name “Evusheld”, was the first available pre-exposure prophylaxis for COVID-19 other than vaccination. It received an EUA from the FDA after sufficient trial data showed efficacy in preventing SARS-CoV-2 infections and subsequent severe disease. Its potential benefits for high-risk immunocompromised patients generated a lot of interest. Individuals with multiple myeloma fall into this category, as they are characterized by attenuated immune responses and, in some cases, vaccines have limited efficacy. **Methods:** this single-center, prospective study included consecutive patients with multiple myeloma. All individuals were considered high-risk for COVID-19 due to their underlying disease. Baseline demographic and clinical characteristics, as well as data regarding COVID-19 infection and antibodies, were collected. Patients were administered two intramuscular 150 mg doses of Evusheld and were monitored during the follow-up period. **Results:** one hundred and eleven multiple myeloma patients were included in this analysis, with a median age of 64 years (range 58–69) and fifty-three were females (47.7%). Fourteen patients (12.6%) had a prior history of COVID-19 and all patients were vaccinated with either three or four doses of mRNA-based vaccines. An increase was observed in the median neutralizing-antibody levels before and after tixagevimab/cilgavimab administration, from 92.6% to 97.3%. The high levels were sustainable, with a median neutralizing-antibody level of 95.4% at 3 months post Evusheld administration. Overall, nine patients (8.1%) were diagnosed with COVID-19 during the follow-up period, at a median of 31 days. There were no SARS-CoV-2- infection-related hospitalizations or deaths. The monoclonal antibody combination was well tolerated, with no infusion-related reactions or major adverse events, and pain at the injection site only was reported by 33 patients (30%). **Conclusions:** tixagevimab/cilgavimab (Evusheld) seemed beneficial for patients with multiple myeloma, who presented high neutralizing-antibody levels and a low incidence of COVID-19 during the initial Omicron wave. No new safety concerns emerged. However, novel combinations of monoclonal antibodies against the new circulating variants of SARS-CoV-2 are deemed necessary in view of the emergence of immune tolerance.

## 1. Introduction

The outbreak of the Coronavirus Disease 19 (COVID-19) began an era of great challenges in the clinical management of patients with hematological malignancies [1]. In such cases, the immune response to Severe Acute Respiratory Syndrome Coronavirus 2 (SARS-CoV-2) is compromised, thus leading to considerably high morbidity and mortality rates of hematologic patients hospitalized with SARS-CoV-2 infections [2,3].

As patients with hematologic malignancies have lower seroconversion rates after infection or vaccination (especially those treated with anti-CD20 immunotherapies) than the general population, concerns arise about their protection against the virus [4]. 

Such is the case with multiple myeloma (MM) patients, who continue to be vulnerable, with a high risk of hospitalization, severe disease progression and death. Moreover, established anti-myeloma care includes drug combinations with significant hematological toxicity and immune-system impairment. This increases the risk of severe infections [5,6]. Their management either in terms of prophylaxis or treatment remains challenging [7,8].

Vaccination with variant-specific boosters and prevention measures such as mask wearing and avoiding crowded places continue to be the backbone of multiple myeloma patients’ prophylaxis. The timely use of antivirals is also recommended, as they are effective against Omicron subvariants [8,9]. Convalescent plasma, on the other hand, appears to be of low value and only beneficial in certain cases or sub-populations [10].

Monoclonal antibodies, which restore, enhance or mimic the immune system’s response against pathogens, irrespective of its status, are potential candidates for COVID-19 immunoprophylaxis, treatment or management in general [11].

The first pre-exposure prophylaxis product for COVID-19 other than the vaccines is Evusheld, a combination of two monoclonal antibodies (tixagevimab and cilgavimab). They are derived from antibodies isolated from B-cells of individuals infected with SARS-CoV-2. They work by binding to the SARS-CoV-2 spike protein receptor-binding domain in order to potentially neutralize the virus [12,13,14,15]. Studies first showed prophylactic and therapeutic properties in vitro and in non-human subjects [12], followed by positive results in terms of safety and efficacy from clinical trials [16]. This led to an Emergency Use Authorization (EUA) by the Food and Drug Administration (FDA).

By late spring/early summer of 2022, Omicron became the prevailing SARS-CoV-2 strain worldwide, featuring four primary sub-variants: BA.1, BA.2, BA.3, B.A.4 and B.A5. BA.4 and BA.5 bear additional mutations to their spike protein [17]. This raised concerns about their capacity to escape antibodies elicited by the Omicron infection as well as therapeutic monoclonal antibodies that are based on prior SARS-CoV-2 variants [18]. On the 26 January 2023, the FDA revised and withdrew the Emergency Use Authorization for Evusheld, citing reports from the Centers for Disease Control and Prevention (CDC) of dominant Omicron variants against which Evusheld may not be as potent as previously.

Despite this, during the EUA period, Evusheld was widely used in hospital settings and studies. Herein, we present the results of a study on the pre-exposure prophylaxis of high-risk individuals for severe COVID-19 infection due to underlying multiple myeloma, who received tixagevimab/cilgavimab.

## 2. Materials and Methods

Consecutive patients with multiple myeloma were prospectively enrolled at a single institution (Department of Clinical Therapeutics, Alexandra General Hospital, School of Medicine, National and Kapodistrian University of Athens, Greece). The first patient was enrolled on 1 January 2022 and the last follow up date was 31 May 2023. All patients were deemed as high-risk individuals for severe COVID-19 outcomes due to underlying disease. Eligibility criteria included a documented diagnosis of symptomatic multiple myeloma according to the International Myeloma Working Group (IMWG) criteria and a confirmed negative SARS-CoV-2 PCR test.

All enrolled individuals were tested negative for SARS-CoV-2 by polymerase chain reaction (PCR) testing prior to administration of monoclonal antibodies for COVID-19 prophylaxis. Further COVID-19 testing was carried out before the anamnestic dose and also at any time point that was deemed necessary (e.g., if a patient had symptoms suggestive of COVID-19).

Every patient underwent measurement of neutralizing antibodies (NAbs) against SARS-CoV-2 using an FDA-approved methodology (enzyme-linked immunosorbent assay—ELISA, cPass SARS-CoV-2 NAbs Detection Kit; GenScript, Piscataway, NJ, USA) before tixagevimab/cilgavimab injection and at one and three months thereafter.

Baseline demographic and clinical characteristics (age, sex, body mass index (BMI), performance status (PS), international staging system (ISS) indexing, line of treatment, autologous stem cell transplant, COVID-19 illness and vaccination history), as well as neutralizing-antibody levels and patient outcomes, were collected and analyzed.

The levels of the international staging system indexing, denoted as ISS in the baseline clinical characteristics, are defined as:

- **Stage 1**: Sβ2M < 3.5 mg/L; serum albumin ≥ 3.5 g/dL
- **Stage 2**: Sβ2M < 3.5 mg/L; serum albumin < 3.5 g/dL; or β2M 3.5 to 5.5 mg/L, irrespective of serum albumin
- **Stage 3**: Sβ2M > 5.5 mg/L

Evusheld was administered at 150 mg as two intramuscular injections. An anamnestic second dose was scheduled and administered 6 months after the prime dose. Patients were followed for 3-to-6 months post the first tixagevimab/cilgavimab dose and for 1-to-3 months after the second dose. The study was approved by the institutional review board.

## 3. Results

A total of 111 enrolled patients were included in this analysis, who were followed for a median of 5 months (range 3–6 months). The median age was 64 years (range 58–69) and 53 were females (47.7%). 

Patient performance status was distributed as follows: PS 0 (n = 54, 48.6%), PS 1 (n = 46, 41.4%), PS 2 (n = 9, 8.2%), PS 3 (n = 1, 0.9%) and PS 4 (n = 1, 0.9%). The international staging system (ISS) indexing was distributed as follows: 44 patients (39.6%) were ISS 1, 51 patients (46.0%) were ISS 2, and 16 (14.4%) were ISS 3. 

At the time of enrollment, 73 (65.8%) patients were at the first line of treatment, 26 (23.4%) patients were receiving the second line of treatment, and 12 (10.8%) patients were receiving subsequent lines of therapy. A total of 44 (40%) patients had previously undergone autologous stem cell transplant.

At the time of tixagevimab/cilgavimab administration, 30 patients (27%) were receiving combinations including anti-BCMA agents, 33 patients (30%) were receiving combinations including anti-CD38 drugs, and 48 (43%) were on other treatment regimens. 

Fourteen patients (12.6%) had a prior history of COVID-19. As far as vaccination against COVID-19 is concerned, 65 patients (58.6%) had received three doses and 46 patients (41.4%) had received four doses. All individuals were vaccinated with mRNA-based vaccines. Table 1. summarizes the baseline patient characteristics.

The median Nab level (%) before administration of tixagevimab/cilgavimab was 92.6% (IQR 71.3–96.0), whereas it increased to 97.3% (IQR 95.5–97.8) one month thereafter. In total, nine (8.1%) patients were diagnosed with COVID-19 during the follow-up period, at a median of 31 days (IQR 8–55 days). All of these patients received nirmatrelvir/ritonavir (Paxlovid) for 5 days as outpatients along with supportive care, as per standard clinical practice, and recovered completely. There were no SARS-CoV-2-infection-related hospitalizations or deaths.

Long-term results were available only for a subset of patients. Neutralizing-antibody levels (%) remained high at 3 months after administration, with a median of 95.4% (IQR 93.7–97.9) among the 33 patients (30%) tested. Six months post Evusheld, data were very scarce, as they were only available for four patients (3.6%). Among these patients, the median neutralizing-antibody level was 95.1% (IQR 94.9–95.7).

The neutralizing-antibody activity was evaluated in a group of 23 patients (20.7%) before and at one and three months after the anamnestic (second) dose of tixagevimab/cilgavimab. At one month after the second dose, all 23 patients were analyzed. The median neutralizing-antibody levels remained high before and after one month of the booster Evusheld dose. At three months after the second dose, samples were available from only 5 out of the 23 patients. The median neutralizing-antibody level was 95.5% (IQR 95.4–95.6). 

Tixagevimab/cilgavimab was well-tolerated; no infusion-related reactions or major adverse events were reported. Thirty-three patients (30%) experienced pain at the injection site that resolved after a few days.

Table 2 summarizes the results from neutralizing-antibody and reaction assessments at each timepoint.

## 4. Discussion

In our study, which enrolled patients with multiple myeloma, tixagevimab/cilgavimab seemed beneficial as a pre-exposure prophylaxis measure against SARS-CoV-2. Among these high-risk patients, there was a very low incidence of COVID-19 occurrence, while increased and sustained neutralizing-antibody levels were also recorded during the follow-up period.

Tixagevimab/Cilgavimab (Evusheld) generated high interest initially, being the first available pre-exposure prophylaxis product for COVID-19, other than vaccines, with promising potential. A randomized, placebo-controlled trial, enrolling adults with an increased risk of inadequate immune response to COVID-19 vaccination or exposure to SARS-CoV-2 (or both), reported that a single dose of tixagevimab/cilgavimab had beneficial protective effect against COVID-19, with no safety concerns [19]. A large-scale meta-analysis involving 27,932 patients enrolled in various studies demonstrated benefits from Evusheld administration, as there was a statistically significant decrease in the risk of progression to severe COVID-19 disease (e.g., decreased hospitalization, oxygen need, ICU admission, etc.) [20]. A two-arm comparative study carried out among members of the Maccabi Health Care Services also recorded lower infection, hospitalization and mortality rates [21]. While unvaccinated individuals initially benefited, this cannot safely be assumed as of now, since variants have changed and vaccination is widespread [22].

Its potency was even more significant and sought-after for immunocompromised patients who are unable to mount a sufficient immune response after vaccination and thus remain at high risk of severe COVID-19 disease. Due to the illness itself and the therapeutic modalities, patients with hematologic malignancies, such as multiple myeloma, leukemia and lymphoma, often have an impaired immune system. Several underlying factors may be responsible for this. Hematologic cancers may directly inhibit the development and function of immune cells, including T cells, B cells, and natural killer (NK) cells. Cancer cells also produce cytokines and chemokines that suppress the maturation of immune cells. These immune cells are essential for building a successful immunological response to diseases and vaccinations. Hematologic malignancies typically affect the bone marrow and promote an immunosuppressive microenvironment [23]. 

Treatment regimens that include chemotherapy are commonly used in individuals with hematologic malignancies. Chemotherapy targets rapidly proliferating cells, such as immune and cancer cells. Such therapies may lower immune-cell counts and function, further impairing the immunological response [24]. Furthermore, some patients may undergo stem cell transplantation as part of their therapy for the underlying hematological cancer. During this procedure, the immune system is severely compromised due to the high-dose chemotherapy before it recovers and restores its full functionality, which may take several months. Apart from chemotherapy, immune-modulating agents including immunosuppressive drugs to prevent stem cell transplant rejection or treat graft-versus-host disease (GVHD) may further impede immune response [25]. 

Last but not least, the patient’s age and general health status may also affect their ability to mount a successful immune response. The immune system of older individuals and those with pre-existing medical problems may be less robust, which may impact seroconversion rates [26].

A review involving real-world and trial data concluded that the monoclonal antibody combination was successful in lowering hospitalization and mortality rates in vulnerable populations. The prophylaxis effectiveness of the antibody combination in reducing the incidence, hospitalization, and mortality linked to COVID-19 in solid-organ transplant recipients, patients with immune-mediated inflammatory diseases and hematological malignancies, and patients undergoing B-cell-depleting therapies is supported by real-world data. Encouraging results have also been reported after the administration of tixagevimab/cilgavimab in patients with COVID-19 in terms of reducing the risk of severe disease and death when administered promptly after the SARS-CoV-2 infection diagnosis [27]. Another propensity-score-matched analysis yielded statistically significant results, suggesting that the product was effective in reducing the risk of SARS-CoV-2 infection and COVID-19 hospitalization in immunocompromised patients [28]. Real-world data from the administration of tixagevimab/cilgavimab at a large academic center also showed benefits for high-risk individuals who had sub-optimal immune responses to vaccines [29]. Recipients of allogeneic hematopoietic stem cell transplantation during the Omicron wave, a high-risk group, also benefited from Evusheld [30]. Finally, an observational multicenter cohort study of severely immunocompromised patients receiving tixagevimab/cilgavimab as pre-exposure prophylaxis reported low infection rates and severe illness occurrences [31]. Regarding safety, the benefit of an additional protection against COVID-19 outweighs the risk of potential adverse events, which are anyway limited [32]. 

Patients with hematological malignancies are a large percentage of the immunocompromised populations, and early study cohorts showed statistically significant protective benefits from the Evusheld administration against COVID-19 [33]. Multiple myeloma patients form a large sub-population of the aforementioned immunocompromised patients with hematological malignancies. Their management is challenging, as their immune system is compromised due to their underlying disease and therapies against myeloma. This became even more evident during the pandemic. COVID-19 is considered a multisystemic disease, and numerous studies have shown persistent or late-onset symptoms, including post-disease hematological disorders [34]. Patients with multiple myeloma are at high risk of infections with severe outcomes [35,36]. 

The emergence and widespread availability of anti-SARS-CoV-2 vaccines was of great significance and formed an important preventive strategy, but it was not without limitations [37]. A study including older multiple-myeloma patients showed low neutralizing antibody responses after the first BNT162B2 vaccine dose [38]. A follow-up study showed that vaccination with either two doses of the BNT162b2 or one dose of the AZD1222 vaccine leads to a lower production of neutralizing antibodies in multiple-myeloma patients compared to healthy individuals [39]. In addition, the same study showed that the immune response to the aforementioned vaccines was dependent on the active treatment for the underlying disease. Specifically, treatment with anti-CD38 monoclonal antibodies or belentamab mafoditin were independent prognostic factors for a low humoral response to vaccination [40]. Then, after the prevalence of the Omicron strain, significantly lower neutralizing-antibody responses to the BA.4/5 variants were recorded in patients, including those with hematological malignancies [41]. Such observations, at different time stamps of the pandemic, underlined the necessity for booster doses [42,43].

All the above highlight the importance of preventive strategies with measures for pre-exposure protection to COVID-19 for multiple-myeloma patients [44]. As such, the availability of a potentially effective new product seen in Evusheld generated high interest for the management of patients with hematological malignancies and thus multiple myeloma. A study that enrolled multiple-myeloma patients in order to assess the efficacy of tixagevimab/cilgavimab as a pre-exposure prophylaxis measure concluded that it can offer better protection than vaccination alone in preventing COVID-19 infections or disease severity [45]. A retrospective analysis of medical records of patients with B-cell malignancies also showed benefits from pre-exposure prophylaxis with tixagevimab/cilgavimab, as low hospitalization rates and no COVID-19-related deaths were recorded [46]. Potential protective benefits were also suggested after a case report on patients with hematological malignancies who received the tixagevimab/cilgavimab combination [47]. A trial involving 203 patients with hematological malignancies who consented to receive Evusheld reported infrequent COVID-19 infections and hospitalizations [48].

A retrospective study evaluating the pre-exposure prophylaxis efficacy of tixagevimab/cilgavimab for patients with hematological disorders (including malignancies) during the Omicron era in Japan showed benefits from its administration, as COVID-19 incidence and critical disease occurrence were low [49]. The importance of continued monitoring and strategy adaption to new variants and conditions was stressed. A systematic review and meta-analysis of the protective properties of the monoclonal antibody combination, including patients with hematological malignancies in the Omicron era, also highlighted its clinical effectiveness [50]. A 6-month prospective study on 31 immunocompromised patients receiving immunosuppressive therapy reported that Evusheld significantly reduced the severity of breakthrough COVID-19 infections during the BA.4 and BA.5 Omicron wave [51]. A single-center retrospective study analyzing clinical outcomes of patients with hematological malignancies who were administered tixagevimab/cilgavimab for COVID-19 prevention or treatment generated beneficial results [52]. An additional retrospective analysis on the serologic responses of 181 patients with hematological malignancies failed to show significant associations between seroconversion and COVID-19 infection, but reported that no patient who received tixagevimab/cilgavimab died [53]. On the other hand, low neutralizing activity against the current Omicron variants in patients with B-cell malignancies was also reported [54]. In another retrospective observational study on patients with hematological malignancies, lower COVID-19 infection rates were reported but there was no significant difference in disease severity or hospitalization rates [55].

Tixagevimab/cilgavimab has been also evaluated in other individuals at high risk of COVID-19. Thomas et al. conducted a multicenter observational study including 115 patients with inflammatory or autoimmune diseases who were receiving immunosuppressive therapy such as the anti-CD20 monoclonal antibody rituximab, methotrexate and corticosteroids. After a median follow-up of 4.3 months, 23 COVID-19 cases were reported. The authors noted that the incidence of COVID-19 in this population was lower than the COVID-19 incidence in the general population during the study period, whereas patients who received an anamnestic injection had better protection against SARS-CoV-2 infection [56]. Another small study included patients with glomerular diseases who were under treatment with rituximab. A total of 22 patients received tixagevimab/cilgavimab and were prospectively compared with 28 patients who did not receive monoclonal antibodies as prophylaxis for COVID-19. During the follow-up period (mean 3.7 months), no COVID-19 cases were reported among patients who received Evusheld compared to a 39% incidence of SARS-CoV-2 infections among the others [57].

The emergence and prevalence of the Omicron strain and its subsequent sub-variants hindered Evusheld’s efficacy, as various studies and analyses showed decreased potency against them [58,59,60]. Mixed results were reported regarding which Omicron lineages the monoclonal antibodies are potent against [60,61,62]. For example, a serological analysis of samples collected by a trial on immunocompromised patients suggested relevant activity of tixagevimab/cilgavimab against BA.4/5 but minimal activity, on the other hand, against BQ.1.1 and XBB.1 [63]. Resistance of Omicron subvariants BA.2.75.2, BA.4.6, and BQ.1.1 to neutralizing antibodies was also reported [64]. From a physicochemical point of view, it has been also shown that the novel Omicron variants have a high number of positively charged residues, which in turn attenuate the electrostatic attraction between Evusheld and viral RBD [65]. This raises concerns, as neither is consensus reached, nor are there sufficient trial data on Omicron subvariants available. Eventually, the FDA withdrew the EUA for Evusheld, and as of 26 January 2023 it is not authorized for use in the U.S. [66]. The rapid evolution of SARS-CoV-2 poses significant challenges in the development and delivery of novel antiviral drugs both for prevention and treatment. Contemporary and adaptive clinical-trial designs are deemed necessary to reduce the timelines. In this context, PANORAMIC is a platform clinical trial based on a master protocol including multiple treatment arms that randomize patients with early-onset COVID-19 to receive the usual care with or without novel antivirals [67]. 

As such, there is no clear and robust evidence regarding the effectiveness or ineffectiveness of tixagevimab/cilgavimab in the Omicron era. While some studies show beneficial effects from its use, others report low neutralizing capabilities and non-significant results. The aforementioned lack of consensus poses a limitation to our study, which enrolled patients in a varying timeline and was concluded before the now-dominant era of newer Omicron sub-variants. In our study, tixagevimab/cilgavimab seemed to be effective in protecting high-risk multiple-myeloma patients from COVID-19 infections and critical disease. Long-term data were limited, as neutralizing antibody testing after 3 or 6 months was only carried out on a sub-group of the initial population. The anamnestic dose was also administered in a small group of patients, thus resulting in limited data to draw firm conclusions. The aforementioned low sample size also poses as a limitation to our study and thus to our concluding secondary outcomes. 

On the other hand, the novelty of this study is worth noting, as its long follow-up period enables us to study the evolution of antibody titers in patients with multiple myeloma treated with tixagevimab/cilgavimab as prophylactic therapy for COVID-19. Moreover, it provides real-world evidence of a particularly vulnerable population enrolled during the EUA period spanning a long time period in the Omicron era.

## 5. Conclusions

As there was a very low incidence of COVID-19 occurrences among high-risk patients with multiple myeloma in our study cohort, pre-exposure prophylaxis with Tixagevimab/Cilgavimab (Evusheld) showed a potential benefit for this patient group. Increased and sustained high levels of neutralizing antibodies were recorded post administration of the first and booster dose, with no safety concerns. Additional data, preferably from randomized controlled trials, on the current or future state of the pandemic, would help determine if Tixagevimab/Cilgavimab can be effective for COVID-19 prophylaxis in patients with multiple myeloma in the era of currently dominant SARS-CoV-2 variants. Adaptations within the “antibody cocktail” according to the circulating variants of interest might be necessary to maintain the optimal efficacy.

## Figures and Tables

**Table 1:** Patient characteristics at baseline.

Variables	Measurements
Age—years (median, IQR)	64 (58–69)
Age group—no. (%)	
<50	8 (7.2%)
≥50	103 (92.8%)
≥60	75 (67.6%)
≥70	24 (21.6%)
Female sex—no. (%)	53 (47.7%)
BMI—kg/m^2^ (median, IQR)	26.1 (23.0–27.9)
PSS group—no. (%)	
PS 0	54 (48.6%)
PS 1	46 (41.4%)
PS 2	9 (8.2%)
PS 3	1 (0.9%)
PS 4	1 (0.9%)
ISS group—no. (%)	
ISS 1	44 (39.6%)
ISS2	51 (46.0%)
ISS3	16 (14.4%)
Line-of-treatment group—no. (%)	
1st line	73 (65.8%)
2nd line	26 (23.4%)
3rd/4th/5th line	12 (10.8%)
MM-treatment-type group—no. (%)	
Anti-BCMA agents	30 (27%)
Anti-CD38	33 (30%)
Other	48 (43%)
Autologous stem cell transplant—no. (%)	44 (40%)
Prior history of COVID-19—no. (%)	14 (12.6%)
Vaccine doses against COVID-19—no. (%)	
3	65 (58.6%)
4	46 (41.4%)

**Table 2:** Neutralizing-antibody activity at each timepoint.

Result	Measurement
NAbs pre Evusheld—% (median, IQR) Sample size—no. (%)	92.6% (71.3–96.0)
NAbs post Evusheld—(% median, IQR) Samples—no. (%)	97.3% (95.5–97.8)
COVID-19-positive post Evusheld—no. (%) Timeline—days (median, IQR)	9 (8.1%) 31 (8–55)
NAbs 3 months post Evusheld—% (median, IQR) Sample size—no. (%)	95.4% (93.7–97.9) 33 (30%)
NAbs 6 months post Evusheld—(% median, IQR) Sample size—no. (%)	95.1% (94.9–95.7) 4 (3.6%)
NAbs pre second Evusheld dose—% (median, IQR) Sample size—no. (%)	95.1% (94.8–95.9) 23 (20.7%)
NAbs post second Evusheld dose—% (median, IQR) Sample size—no. (%)	95.1% (94.9–96.0) 23 (20.7%)
Infusion-related reactions—no. (%)	0 (0%)
Major adverse events—no. (%)	0 (0%)
Pain at the injection site—no. (%)	33 (30%)

## Data Availability

Data available upon request from the corresponding author.
